# Transformational entrepreneurship and its effect on readiness for change, psychological capital, and employee performance: evidence from an Indonesian bank

**DOI:** 10.12688/f1000research.52480.1

**Published:** 2021-09-03

**Authors:** Michael Gunawan, Retno Wijayanti, Febri Nila Chrisanty, Budi W. Soetjipto, Ani Wahyu Rachmawati, Santi Rahmawati

**Affiliations:** 1 University of Indonesia, Depok, West Java, 16424, Indonesia; 2 Universitas Wanita Internasional, Bandung, West Java, 40173, Indonesia; 3 Research Synergy Foundation, Bandung, West Java, 40291, Indonesia

**Keywords:** transformational entrepreneurship, readiness to change, psychological capital, employee performance, structural equation model, banking industry

## Abstract

Continuing failures of financial capitalism across borders have led corporation to develop a more balanced economic growth model of transformational entrepreneurship that emphasises both short-term economic and longer-term social impacts. The model encourages entrepreneurial activities that bring major changes in the related markets and industries, as well as changes in society and culture. At the corporate level, transformational entrepreneurship prepares employees for any potential changes induced by a dynamic environment; it also improves the psychological capital of individual employees, and effective transformational entrepreneurship can eventually accelerate performance. The purpose of this study is to investigate (1) the direct and indirect effects of transformational entrepreneurship on readiness for change, psychological capital and employee performance, and (2) how the effects to readiness for change and psychological capital influence employee performance. The study data were collected using questionnaires completed by employees in 257 branches of a state-owned bank with locations throughout Indonesia. The data were analysed using the structural equation model. The results show that transformational entrepreneurship significantly and positively influences readiness for change, psychological capital, and employee performance and that readiness for change and psychological capital significantly and positively influences employee performance. Additionally, the effect of transformational entrepreneurship on employee performance is more significant if it is related to psychological capital than to readiness for change or to aspects of employee performance unrelated to transformational entrepreneurship. These findings enrich our understanding of transformational entrepreneurship and its value related to the direct and indirect effects on variables such as readiness for change, psychological capital and employee performance.

## Introduction

To sustain viability in a dynamic and turbulent environment, a company needs transformational leaders with high level of integrity and willingness to extend their employees’ efforts to achieve targets, which, in turn, will enhance the company's performance (Lafley, 2006). These characteristics closely relate to entrepreneurial qualities such as being innovative, proactive, and willing to take risks ( Covin & Slevin, 1991; Miller & Friesen, 1982; Slevin & Covin, 1991). Bass (1985) and others (e.g., Howell & Avolio, 1993; Howell & Higgins, 1990) have suggested that transformational leadership is related to innovation in the organisation, while Bateman and Crant (1993) and others (e.g., Crant, 2000; Deluga, 1998) have found that transformational leadership is linked to proactive behaviour, an aspect of entrepreneurship.

As mentioned above, success in dealing with uncertain and unpredictable environments lies not only in survival but also in sustainability. For that reason, the company needs both (shorter-term) economic/financial result and (longer-term) social impacts, so the success of the company can cascade into the wealth of the society (Maas, Jones & Lockyer, 2019). A wealthy society has the economic power to support the company, continually allowing for the company's continuous growth into the future. Entrepreneurs have tried to accommodate such social impacts by developing the concept of social entrepreneurship. However, social entrepreneurship is more focused on the work of community, voluntary, and public organisations, as well as private firms working for social rather than for-profit objectives ( Shaw & Carter, 2007, p. 419). Thus, “its impact has been limited to date as its solutions are rarely devised with scalability and true economic sustainability in mind” ( Marmer, 2012, p.2).

Hence, there is a need for entrepreneurship that can successfully transform profit-motivated institutions. The concept of transformational entrepreneurship has been introduced, going beyond just combining transformational leadership and entrepreneurship. It encourages entrepreneurial progress by combining individuals, communities, and institutions that interact and collaborate to take advantage of current opportunities on a broader scale ( Maas, *et al.*, 2019; Schoar, 2010). Moreover, transformational entrepreneurship encourages entrepreneurial activities that bring major changes in the market and industry, as well as in social and cultural life ( Marmer, 2012).

Embracing economic results and social impacts in its goals, the company must shift its perspective from an economic to a socio-economic standpoint. The shift requires the company and its employees to be ready for change. Being ready for change means the employees of such a company are dedicated and capable of implementing necessary change, through commitment and efficacy ( Weiner, 2009). Moreover, embracing a socio-economic perspective means putting more emphasis on sustainability rather than mere survival. The socio-economic perspective gives employees hope for future success, growth optimism, and confidence (self-efficacy). Despite the challenges and adversities that employees are likely to face, they can persevere and remain resilient enough to succeed.

Hope, optimism, self-efficacy, and resilience are components of psychological capital (Luthans, Youssef & Avolio, 2018). As previously mentioned, the objective of adopting transformation entrepreneurship is for the company to sustain growth through continuously increasing employee performance. Referring to Pradhan and Jena (2017), employee performance includes task performance (performance related to the job description and/or employment contract), adaptive performance (performance related to dynamic work situations, such as technological changes), and contextual performance (performance related to maintaining and developing a team spirit). According to a review of the past literature, and to the best of the authors’ knowledge, there is a lack of research connecting transformational entrepreneurship to organisational readiness for change, psychological capital, and employee performance. Therefore, examining such organisational factors in relation to transformational entrepreneurship is the objective of this study.

Previous research does indicate that both organisational readiness for change and psychological capital can relate to employee performance. Weeks *et al.* (2004) found a positive and significant association between organisational readiness for change and job performance in sales organisations. Job performance examined in their study was essentially viewed as task performance. Meanwhile, Gooty *et al.* (2009) found a positive and significant association between psychological capital and job performance in an educational institution. They examined performance as in-role and extra-role performance, which refers to task and contextual performance. Peterson *et al.* (2011) also found that psychological capital was positively related to change in supervisor-rated performance and change in objective performance in a financial service organisation. Both of those performance categories are considered task performance. In short, there have been limited studies that connect both organisational readiness for change and psychological capital to performance, particularly simultaneously connecting to task performance, adaptive performance, and contextual performance. Consequently, examining such connections is the objective of this study.

This study took place in a large, state-owned bank in Indonesia. The business of banking was specifically chosen because it has long been known as a very competitive landscape, yet the industry is highly regulated. To compete successfully, each bank must be innovative, proactive, and risk-taking but remain within the boundaries of government laws and regulations. The emergence of digital technology has made the competition harsher than ever before, particularly with the presence of some tech start-ups that are able to launch without the support of banks. In other words, the banking industry is transforming itself into a more digital-savvy industry.

On the heels of the digital revolution, a transformational entrepreneurship within the banking industry is essential for banks to simultaneously enhance their economic and financial performance and increase positive social impacts by extending the scope of banking operations and, in turn, touching the lives of many more people. Individual bank branches comprise the frontline for banks’ transformational entrepreneurship practices. These are the business units that acquire and maintain customers for future revenue generation and engage with the local communities to create and maintain their social impacts. Such impacts are particularly crucial in a country like Indonesia, with more than 250 million people spread out over more than 17,000 islands. The banks that can create social impacts will generate wealth throughout the country, and this wealth will be fed back to the banks in terms of financial income. If successful, the wealth of the world's fourth most populous country will be more than enough to sustain banks’ growth for a very long time.

### Transformational leadership, entrepreneurship, and transformational entrepreneurship

According to Bass (1985) and Burns (2003), a transformational leader is one who can affect the evolution and growth of the character of an organisation, bringing it to a new, desired condition through dramatic changes. The leader can inspire their followers with challenging but achievable future vision and objectives. Moreover, as a role model, the leader can continuously motivate followers and intellectually encourage them to always look for creative and innovative ways to solve every problem they encounter. A transformational leader also often dedicates time to be with their followers to listen to their views, opinions, and feedback and provide guidance, support, and direction to take advantage of the opportunities that may arise.

Meanwhile, Shane and Venkataraman (2000) defined entrepreneurship as individuals and processes that led to the discovery, evaluation, and exploitation of opportunities. Hsieh, Nickerson, and Zenger (2007) further argued that these opportunities existed or were created to fill the organisation's needs and wants. Such opportunities are then evaluated based on the organisation's desirability and feasibility before being exploited (McMullen & Shepherd, 2006; Shane & Venkataraman, 2000). A transformational leader can inspire, motivate, and guide their followers to be entrepreneurs. The leader first clarifies the future vision and objectives, then points out the gaps between the existing conditions and those necessary to achieve the vision by engaging in entrepreneurial behaviour. Along the way, a transformation leader urges his or her followers to be creative and innovative in identifying and creating opportunities.

A transformational leader can extend his or her inspiration to include economic results and social impacts aligned with the organisation's vision statement and objectives. In this case, the leader is said to practice transformational entrepreneurship ( Schoar, 2010; Virmani & Lepineux, 2016; Jones & Maas, 2019). Being a role model, a transformation leader expects his or her followers to be transformational entrepreneurs. They each have the basis of an entrepreneur but with greater concern for the well-being of society where their organisation operates and to which it serves. Removed from them is the ‘winner-takes-all’ mentality; instead, they operate with a ‘let’s-grow-together’ mentality. Basing their quest for opportunities using this new mentality can result in totally different solutions for filling in gaps and manifesting the desired future conditions. Their organisations may be more sustainable as they receive strong support from the society encompassing the organisation.

### Hypotheses development

The successful inclusion of social impacts in an organisation's vision and objectives requires different behaviours. The behaviours’ essence is still entrepreneurship, but this entrepreneurship goes beyond merely finding and taking advantage of opportunities. Transformational entrepreneurship is expected to change and improve neighbouring communities. Prior to exerting actual effort to change such communities, however, the organisation and its employees must commit to it and be capable of implementing the necessary change. According to Weiner (2009), if an organisation and its employees already have change commitment and change efficacy, they are considered ‘ready’ for change. Armenakis, Harris and Mossholder (1993) further emphasised that this readiness must be collective and active, meaning that the whole organisation must be ready to participate in change. Correspondingly, we anticipated that:

*Hypothesis (hereafter H) 1: Transformational entrepreneurship positively affects organisational readiness for change*

The heart of entrepreneurship is opportunity. In transformational entrepreneurship, opportunities have a much wider scope, as an organisation touches the social and cultural lives of entire communities to gain their strong support. When carried out effectively, transformational entrepreneurship inspires the employee's hope and optimism about the organisation's sustained success. Moreover, obtaining a great deal of support from surrounding communities instils employees’ confidence and efficacy - they believe in their ability to succeed. Thereafter, if they encounter obstacles and adversities, they will show resiliency until they get over them. According to Luthans, Youssef, and Avolio (2018), hope, optimism, efficacy, and resiliency are the cornerstones of psychological capital. We, therefore, hypothesised the following:

*H2: Transformational entrepreneurship positively affects psychological capital*

When leaders practise transformational entrepreneurship effectively, their organisation can mobilise strong support from the communities (Maas, Jones & Lockyer, 2015; Sautet, 2013; Schoar, 2010). The support is a return favour for the organisation's improvement that can bring social and cultural life of the communities and be a potential economic power that can support back the organisation ( Virmani and Lepineux, 2016). This economic power can be in the form of loyal customers who repeatedly buy or use organisation's products. These organisation's enthusiasts always speak highly of the organisation and its products. With this support at hand, the organisation and its employees will likely increase their performance. Accordingly, the below hypothesis is proposed:

*H3: Transformational entrepreneurship positively affects employee performance*

In addition, Weeks *et al.*, (2004) demonstrated a positive association between organisational readiness for change and employees’ performance. Employees who perceive the organisation for whom they work are ready to counter changes in the environment and believe that their organisation is committed to upgrading their capability to overcome any possible challenges and obstacles derived from change. These are the challenges and obstacles that can also impede their performance. In other words, organisations perceived to be ready for change helps and supports its employees to get over such changes and eventually perform. In conjunction with the previous notions, we formulate the following hypothesis:

*H4: Organisational readiness for change positively affects employee performance*

Furthermore, scholars like Peterson *et al.*, (2011) and Luthans *et al.* (2007) found a positive relationship between psychological capital and employee performance. These findings are understandable, considering that employees with higher psychological capital use positive thinking have positive attitudes toward work. Thus, they are highly motivated and will exert great efforts to achieve their targets. Consistent with the above findings, we derived a further hypothesis as follows:

*H5: Psychological capital positively affects employee performance.*

## Methods

### Study design and participants

This study uses a causal research design to assess the potential impact of transformational entrepreneurship on organisational readiness to change and psychological capital to produce employees’ superior performance. This study also follows the STROBE guidelines/checklist for cross-sectional studies.

Initially, 462 bank branches were targeted. Our email to potential respondents consisted of a cover letter and a set of questionnaires. Follow-up telephone calls and emails were made to increase the response rate. If the written consent from respondents was not obtained, they were unable to participate in the study. The data from bank managers and their immediate subordinates were collected in April through May of 2020.

To access these employees for data collection, we received permission from the bank's board of directors. They provided their oral and written consent due to the good relationship between the researchers and the Board of Director (BoD) of the state-owned bank. Additionally, because one of the co-authors is a member of the BoD of a state-owned bank, we were permitted to do the research and conduct the survey in the bank's branches. However, the respondents involved were not informed that one of the BoD involved in the study. In addition, the survey responses were kept anonymous to ensure there is no conflict of interest in this research.

### Data collection

We collected data using a structured questionnaire adapted from previous research: Maas *et al*. (2019), Marmer, M. (2012), Schoar (2010), and Virmani and Lepineux (2016). We back-translated the questionnaire from English to Indonesian, but we also included both English and Indonesian in the questionnaire. A copy of the distributed questionnaire can be found in the *Extended data* ( Gunawan *et al*., 2021b). Our respondents were branch managers of a state-owned bank and their immediate subordinates. The respondent's criteria are: 1) Active branch managers who already worked and served in the bank for more than 15 years; 2) Two persons (subordinates) who are directly under the branch managers’ chain of command and already worked in various assignment for the bank. Although the respondents are highly educated and understand English, they still had a better grasp of Indonesian. If they were confused by the language in any item, they could always refer to that particular item in the other language. We had language and industry experts check each item in the questionnaire during its development process to minimise confusion for participants.

Before distributing the questionnaire to the actual respondents, it was pre-tested with branch managers and their immediate subordinates from another bank. Based on the pre-test feedback, we refined and modified the wording of some items to ensure the reliability and validity of each variable meets the required standard. Survey items are answered using a Likert-type scale from 1 (strongly agree) to 7 (strongly disagree).

The items for the transformational entrepreneurship variable were adapted from Maas *et al.* (2019), Marmer, M. (2012), Schoar (2010), and Virmani and Lepineux (2016). This part of the survey includes three categories and 17 items. The categories are ‘Quality of Human Capital’ (six items). ‘Taking Risks and Opportunities in a New Market’ (six items), and ‘Evaluating Changing Conditions’ (five items)”.

The items for readiness for change variable were adopted from Armenakis *et al.* (1993). These items asked the respondents about the organisation's and its people's readiness for change, using terms such as ‘aligned business goals’, ‘improved job security and quality’, ‘trust’, ‘good communication’, ‘sufficient money, and training’, and ‘physical infrastructure availability’.

The items for the psychological capital variable were adopted from Luthans *et al.*, (2018). These items asked the respondents about their hope, optimism, self-efficacy, and resiliency. The items for employee performance variable were adopted from Pradhan and Jena (2017). These items asked the respondents about their employees’ tasks and, adaptive and contextual performances.

### Measure for validity and reliability

This study follows a two-step statistical approach to testing the hypotheses by analysing the outer measurement model and the inner-structural model. First, we conducted a confirmatory factor analysis and dropped items with standardised factor loading below 0.50, which means such items are not valid for the respective construct (Flynn *et al.*, 2010). The results provide the standardised factor loadings for the outer model ranging from 0.570 to 0.922. Next, we examined reliability. Table 1 shows that composite reliability (CR) values range from 0.84 to 0.95, while the Average Variance Extracted (AVE) values range from 0.51 to 0.74 suggesting reliability in our measurement model. Please note that one dimension (adaptive performance) in EP was dropped due to its standardised factor loading < 0.50.

**Table 1.** Standardised factor loading, composite reliability, and average variance extracted.

Latent variable indicator	Standardised factor loading (SFL)*	Reliability CR dan VE*
TE_QHC		CR = 0.92; VE = 0.67
TE_QHC1	0.689	
TE_QHC2	0.800	
TE_QHC3	0.829	
TE_QHC4	0.779	
TE_QHC5	0.922	
TE_QHC6	0.879	
TE_RTT		CR = 0.84; VE = 0.51
TE_RTT01	0.665	
TE_RTT02	0.657	
TE_RTT03	0.720	
TE_RTT04	0.752	
TE_RTT05	0.764	
TE_ECC		CR = 0.94; VE = 0.77
TE_ECC1	0.864	
TE_ECC2	0.917	
TE_ECC3	0.880	
TE_ECC4	0.894	
TE_ECC5	0.841	
RC		CR = 0.92; VE = 0.57
RC_1	0.672	
RC_2	0.848	
RC_3	0.860	
RC_4	0.744	
RC_5	0.740	
RC_6	0.832	
RC_7	0.570	
RC_8	0.797	
RC_9	0.675	
PC_H		CR = 0.93; VE = 0.68
PC_H1	0.898	
PC_H2	0.857	
PC_H3	0.751	
PC_H4	0.870	
PC_H5	0.791	
PC_H6	0.774	
PC_O		CR = 0.94; VE = 0.73
PC_O1	0.862	
PC_O2	0.805	
PC_O3	0.884	
PC_O4	0.879	
PC_O5	0.846	
PC_O6	0.839	
PC_R		CR = 0.87; VE = 0.57
PC_R1	0.797	
PC_R2	0.835	
PC_R3	0.591	
PC_R4	0.780	
PC_R5	0.761	
PC_SE		CR = 0.93; VE = 0.68
PC_SE1	0.748	
PC_SE2	0.696	
PC_SE3	0.855	
PC_SE4	0.853	
PC_SE5	0.879	
PC_SE6	0.889	
EP_TP		CR = 0.93; VE = 0.72
EP_TP1	0.847	
EP_TP2	0.839	
EP_TP3	0.845	
EP_TP4	0.847	
EP_TP5	0.863	
EP_CP		CR = 0.95; VE = 0.74
EP_CP1	0.339 (Not Valid)	
EP_CP2	0.820	
EP_CP3	0.870	
EP_CP4	0.882	
EP_CP5	0.896	
EP_CP6	0.833	
EP_CP7	0.846	
EP_CP8	0.860	
EP_AP		CR = 0.84; VE = 0.52
EP_AP1	0.719	
EP_AP2	0.882	
EP_AP3	0.720	
EP_AP4	0.631	
EP_AP5	0.627	
JC_AT		CR = 0.88; VE = 0.52
JC_AT1	0.697	
JC_AT2	0.692	
JC_AT3	0.749	
JC_AT4	0.537	
JC_AT5	0.656	
JC_AT6	0.853	
JC_AT7	0.801	
JC_C		CR = 0.93; VE = 0.72
JC_C1	0.805	
JC_C2	0.855	
JC_C3	0.880	
JC_C4	0.907	
JC_C5	0.794	
JC_R		CR = 0.91; VE = 0.64
JC_R1	0.840	
JC_R2	0.809	
JC_R3	0.830	
JC_R4	0.635	
JC_R5	0.828	
JC_R6	0.844	
JC_R7	0.412 (Not Valid)	
CWBFS		CR = 0.91; VE = 0.67
CWB_1	0.591	
CWB_2	0.838	
CWB_3	0.854	
CWB_4	0.904	
CWB_5	0.862	

### Structural model analysis

We performed structural path analysis using covariance-based Structural Equation Modelling (CB-SEM) Lisrel 8.80 because we estimated research models with reflective measurement models or factor models ( Rigdon, Sarstedt and Ringle, 2017). Lisrel 8.80 is proprietary software; however, there is alternative free software to perform the statistical such as the R project with the Lavaan package.

### Ethical consideration

This study was approved by Universitas Wanita Internasional Ethical Clearance Committee (Protocol number: 675/SR/WAREK-2/UWI/IV/2020) after due consultation, consent letter had been provided by the researchers to all respondents. The written consent to participate from the head office of a state-owned bank was gained according to document B.738.e-BCU/OTD/LOP/03/2020. The written consent to participate was acquired from respondents through the state-owned bank's Branch Office Heads and Managers. Respondents had provided their consent without any force from anyone. Subsequently, in order to protect the rights and privacy of the respondents, all forms of data were acquired will remain confidential.

## Results

### Structural analysis

Bentler and Chou (1987) suggested that the sample size for CB-SEM must be five times the number of indicators in the research model. The model has 65 indicators, meaning that our sample is supposed to be 325 branches. However, our actual sample is 257 branches, which is below that standard. Consequently, we must simplify the indicators by parcelling ( Rhemtulla 2016) and using latent variable scoring (Joreskog, Sorbom, & Yang-Wallentin, 2006). Next, the second-order confirmatory analysis model (indicators) was transformed parcelled into a first-order confirmatory analysis model (dimension or variable). Parcelling reduced the number of indicators to 50 to make our sample size more than adequate. Parcelling also results in a more stable estimation of parameters for a small sample ( Bandalos, 2002) and enhances the model's fit. The model's fit indicators show that RMSEA = 0.039 (≤0.08**), NNFI = 0.99 (≥0.90**), CFI = 1.00 (≥0.90**), IFI = 1,00 (≥0.90**), RFI = 0.99 (≥0.90**), SRMR = 0.016 (≤0.05**), GFI = 0.97 (≥0.90**), and Norm = 1.38 (≤ 3), which all indicate that the model fits and represents the data.

### Participants

We invited 462 branches of the state-owned bank to participate in the study survey. In the end we received returned responses from 462 branch managers, each from a different branch, and 1404 of the employees under their immediate supervision. Unfortunately, 205 branches returned incomplete responses, so the remaining 257 valid branch's responses provided a response rate of 56%. In this case, 257 valid branch responses are considered complete, meaning that all of them have been filled in by the branch manager and at least 1 (one) out of 2 (two) subordinates of the branch leadership under him. Table 2 provides the respondents’ characteristics.

**Table 2.** Respondent characteristics.

	Branch manager (Team leader)	Officers (immediate subordinates – team member)
Sample size	257	633
Sex	• Male: 96.11% • Female: 3.89%	• Male: 82.46% • Female: 17.54%
Educational background	• Bachelor’s degree: 82.88% • Others: 17.12%	• Bachelor’s degree: 88.94% • Others: 11.06%
Marriage status	• Married: 96.11% • Others: 3.89%	• Married: 94.31% • Others: 5.69%
Age intervals	• <30 years old: 0% • 30-40 years old: 44.36% • >40-50 years old: 39.30% • >50 years: 16.34%	• <30% years old: 0.47% • 30-40 years old: 34.44% • >40-50 years old: 38.86% • >50 years: 26.22%
Working experience	• <6 years: 0.39% • 6-10 years: 15.18% • 11-15 years: 26.46% • 16-20 years: 24.90% • >20 years: 33.07%	• <6 years: 0.47% • 6-10 years: 24.17% • 11-15 years: 15.80% • 16-20 years: 26.86% • >20 years: 32.70%
Tenure in current Branch	• 0-2 years: 91.05% • >2 years: 8.95%	• 0-2 years: 81.52% • >2 years: 18.48%

To assess the potential for non-response bias, we compared the chi-square of responses from early and late respondents (the first and last 20% of responses received). The results indicate there is no significant difference between responses of early and late respondents on key measures.

**Figure 1.**
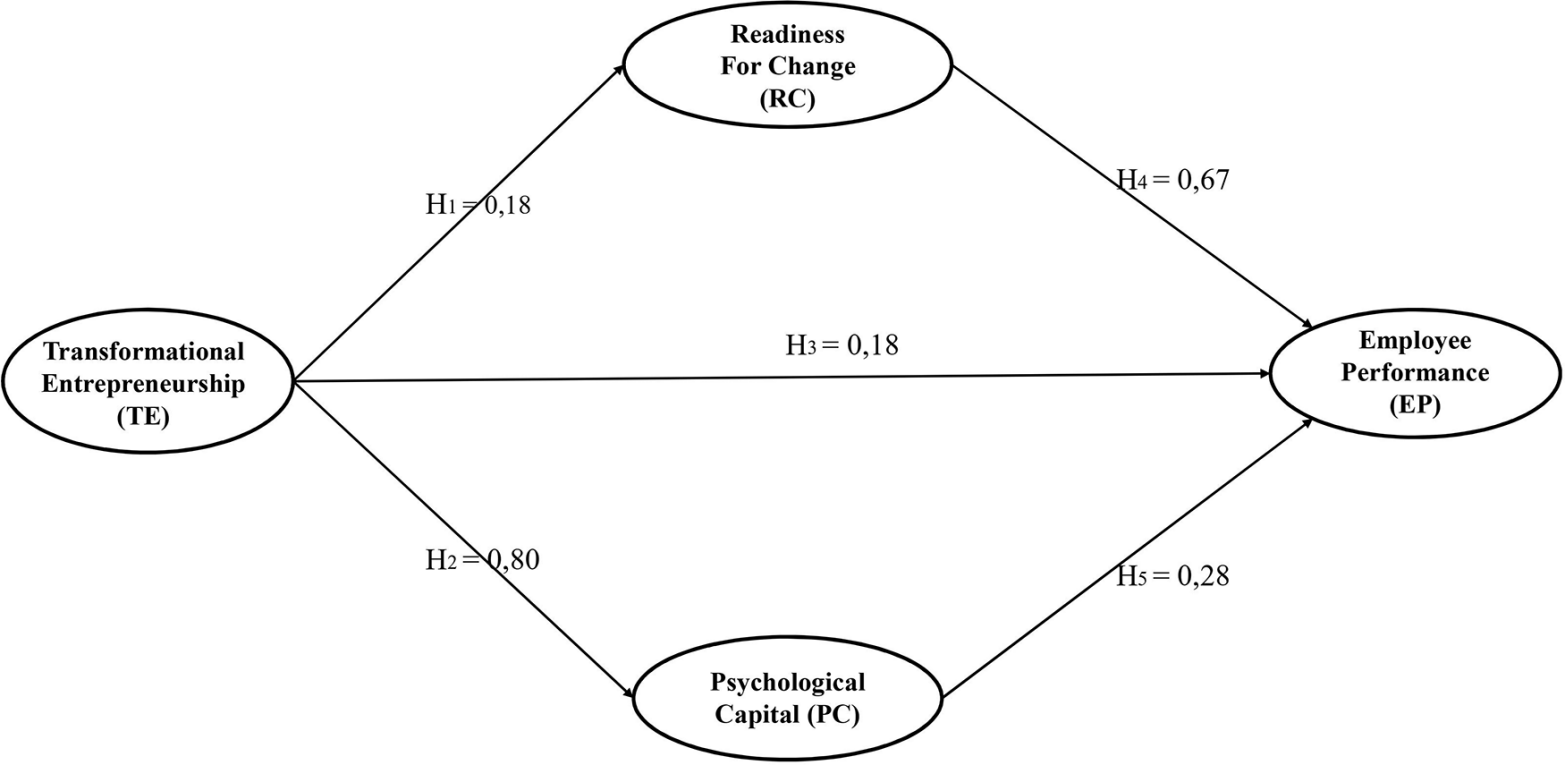
Path diagram of structural research model.

The results of hypotheses testing given in Table 3 and Figure 1 support all the hypotheses. The results support our hypothesis that transformational entrepreneurship has significant positive effects on readiness for change (H1, p < 0.05), psychological capital (H2, p < 0.05), and employee performance (H3, p < 0.05). The path coefficients for the effects of both readiness for change and psychological capital on employee performance are also positive and significant (H4, p < 0.05; H5, p < 0.05). Furthermore, the structural model ( Figure 1) demonstrates the direct and indirect effects of transformational entrepreneurship on employee performance.

**Table 3.** Hypotheses testing result.

Hypothesis	t-Value	Coefficient	Remarks	Summary
H1	2.72	0.18	Significant Positive	Accepted
H2	13.90	0.80	Significant Positive	Accepted
H3	2.42	0.18	Significant Positive	Accepted
H4	15.85	0.67	Significant Positive	Accepted
H5	3.94	0.28	Significant Positive	Accepted

Chi-Square = 31.73, df = 31, P-value = 0,42974, RMSEA = 0.01.

The coefficient for direct effect is 0.18; for the indirect effect through readiness for change, it is 0.1206 (obtained by multiplying the coefficient of transformation entrepreneurship-readiness for change path and the coefficient of readiness for change-employee performance path = 0.18 × 0.67), and the indirect effect through psychological capital is 0.224 (obtained by multiplying the coefficient of transformation entrepreneurship-psychological capital path and the coefficient of psychological capital-employee performance path = 0.80 × 0.28). Based on such coefficients, we can conclude that the biggest effect of transformational entrepreneurship on employee performance was indirectly via psychological capital (0.224 > 0.18 > 0.1206). Nevertheless, in this study, the effect of transformational entrepreneurship on employee performance could be both direct and indirect (partially mediated).

## Discussion

As previously mentioned, transformational entrepreneurship inherits the heart and soul of traditional entrepreneurship. These entrepreneurs are innovative, proactive, and risk-taking in creating and or taking advantage of opportunities in the market ( Covin & Slevin, 1991; Miller & Friesen, 1982; Slevin & Covin, 1990); they also add sustainability for the sake of economic results and social impacts. Support for H1 indicates that with sustainability intact, transformational entrepreneurship can stimulate change within an organisation. Innovativeness, proactiveness, and risk-taking require employees to pursue opportunities even with unclear guidelines. It means employees must tolerate ambiguity, and ambiguity results from a changing environment. In other words, transformational entrepreneurship leads employees to accept that change is inevitable. However, the inevitable change has dual orientations—economic and social—and each has its own dynamics. Whether they like it or not, employees must be ready to acknowledge both dynamics, and they must be committed and able to adapt to them. Therefore, this study's finding enriches the literature on ‘change’ by adding a sustainability perspective, in that one way to achieve sustainability is to support change.

Furthermore, with the emphasis on sustainability, transformational entrepreneurship gives employees hope and optimism for their organisation's longer-term success. Sustainability can gather support from surrounding communities, which, in turn, increases employees’ efficacy to perform and build resiliency to overcome obstacles and adversities. Support for H2 shows that transformational entrepreneurship indeed develops hope, optimism, efficacy, and resiliency, all of the cornerstones of psychological capital (Luthans, Youssef & Avolio, 2018). In addition, Avey (2014) identified four groups of antecedents of psychological capital. They are individual differences, leaders, job design, and pragmatism. Avey found that individual differences (i.e., proactive personality and self-esteem) account for 45% of the variance in psychological capital.

In contrast, leadership (i.e., authentic and ethical leadership) accounts for 32% of such variance. Hence, it is not surprising that we found that transformational entrepreneurship significantly and positively affects psychological capital. Practising transformation entrepreneurship leads employees to be different individuals; they will be more innovative, proactive, and confident in taking risk ( Covin & Slevin, 1991; Miller & Friesen, 1982; Slevin & Covin, 1990). Transformation entrepreneurship also requires strong leadership support (Roth & DiBella, 2015). The findings of this study thus extend our comprehension of transformational entrepreneurship's contribution to the development of psychological capital: it includes individual differences and leadership.

When practising transformational entrepreneurship effectively, an organisation can assemble solid support from communities (Maas, Jones & Lockyer, 2015; Sautet, 2013; Schoar, 2010). Such support is a reciprocal courtesy for the benefits the organisation conveys to them and are a potential economic strength that can sustain the organisation ( Virmani & Lepineux, 2016). This economic strength includes plenty of opportunities the organisation can explore and take advantage of and the communities that are always receptive to, or even excited about, new ideas or new products offered by the organisation. Support for H3 corroborates this notion because to exploit opportunities and deliver new ideas, employees must give their maximum effort based on their ability and consistently do their best. This study's finding enhances our understanding of how to increase employee performance in a socially reciprocal way: it works from the inside out (the organisation provides benefits that impact the society), which leads to an outside-in benefit (the organisation reaps financial rewards from the society in return).

In addition, Weeks *et al.* (2004) found that an individual's perceptions regarding their organisation's readiness for change were significantly and positively related to their job or task performance. Such perceptions reflect their confidence that the organisation will thrive amidst change. This confidence is developed because individuals believe that by implementing change, job quality will be improved. Moreover, individuals can foresee good results from implementing change, hence leading them to understand the value of their performance. As an individual's confidence grows, so does the spirit to take advantage of the change to improve performance. Support for H4 is consistent with these notions. However, this study's finding extends the performance to include contextual performance, which essentially results from pro-social and voluntary behaviour directed at improving the performance of colleagues, teams, and/or the whole organisation ( Pradhan & Jena, 2017). This behaviour goes beyond what is required by the individual's job description or task assignment. In other words, the findings of this study shed light on readiness for change that brings contagious benefits to others in the organisation; these benefits extend beyond any single individuals.

Scholars such as Peterson *et al.* (2011) and Luthans *et al.* (2007) have also found a positive relationship between psychological capital and employee performance. Employees with positive psychological capital usually have high levels of hope, which means having strong determination and conviction about their ability to achieve their goals despite all the hurdles they may face ( Snyder, 2000). Meanwhile, being highly resilient means being able to overcome various unfavourable conditions (Luthans, Avey, Avolio, Norman, & Combs, 2006). Having high levels of (positive) psychological capital also relates to being highly optimistic; regarding their work, this indicates that an employee feels highly positive about their future (Tiger, 1979). Moreover, a high degree of self-efficacy refers to Bandura's social learning theory (1982, 1986), employees’ conviction of their abilities to get their tasks done. Employees who are highly hopeful, resilient, and optimistic and a high degree of self-efficacy are likely to have positive thoughts and attitudes towards work; thus, they have greater motivation and exert more effort to achieve their targets. Support for H5 is consistent with this statement, but psychological capital does not only affect an employee's own performance; the effect extends to the performance of their colleagues, teams, and organisations. This ripple or ‘contagion’ effect occurs because with high hope, resiliency, optimism, and self-efficacy, employees can inspire others; hence, it becomes a source of positivity that spreads psychological capital.

This effect may play a role in increasing the perceived value of psychological capital as an impactful mediator for transformational entrepreneurship and employee performance, as this study has found. Transformational entrepreneurship is characterised by a willingness to tolerate ambiguity and to take risks. It will lead employees to accrue positive psychological capital, particularly in terms of being highly determined to achieve their targets and having strong convictions about their ability to complete their tasks. It is because the employee does not see ambiguity and risks as obstacles. Instead, both are considered natural and, hence, inevitable.

Ambiguity is an integral part of employees’ work, as there can never be full clarity about work, and working with ambiguity has an inherent risk they have to accept. Employees who have positive psychological capital will overcome challenges that comes from ambiguity and risks with their best ability to achieve the goals. Their determination and willingness to give their best effort are likely apparent to others and, thus, become contagious; more and more employees will follow their example. Subsequently, the majority of employees will become highly motivated and put forth their best efforts to achieve their goals. As a result, they will each improve their performance. The findings of this study, therefore, highlight that transformational entrepreneurship with inevitable ambiguity and risks triggers employee's determination and motivation to give their best efforts and seize those challenges. They also highlight how these attitudes are likely to become infectious due to the resulting improvements in employee performance. This infectious role of positive psychological capital may indicate that the direct effects of transformational entrepreneurship on employee performance are less powerful than the indirect effects via psychological capital.

Finally, we are aware that there are some limitations for this research, such as: 1) The research was conducted in the branch office of the state-owned bank. Perhaps in the future, the research also can be done in head office level to gain data from the top management like Board of Directors and Vice President; 2) The study was done in cross-sectional type and the data was collected during the COVID-19 pandemic. The situation is full of uncertainty, so perhaps it may give effect to the result; 3) We got many data from 257 branches of one of the state-owned banks in Indonesia. Perhaps in the future the data can be taken from other state-owned banks in Indonesia. Because currently, Indonesia has four stated-owned banks.

### Future research

We propose three major areas for further research. Firstly, further empirical studies are needed to extend the framework of this study. Variables related to technology, such as digital transformation and technology adoption capability, warrant consideration in future research models. Technology may help organisations reach and engage a broader community to create more and stronger social impacts. Technology has also been demonstrated to change an organisation's business model and products (Hess, Matt, Benlian & Wiesböck, 2016).

Secondly, future research is needed to examine the organisational characteristic of transformational entrepreneurship at the level of the head office of the organisation, as this study was conducted in branch offices. Head office work is mainly at the policy level, so the view and impacts are, theoretically, greater. In addition, future research is needed in industries other than banks. It would be interesting and useful to know how transformational entrepreneurship is practised and how it changes companies in various industries, for example, manufacturing. Other industries’ characteristics are different from those banking, which is considered a service industry. Manufacturing industries produce actual goods, such as clothes, cars, and televisions. Thus, the social impacts and the products are affected by the people.

Thirdly, our research focuses on the consequences of transformational entrepreneurship. Hence transformational entrepreneurship precedes readiness for change and psychological capital when, in reality, their relationships could reciprocate over time. Transformational entrepreneurship, for instance, could be affected by readiness for change and psychological capital. We could not empirically examine reciprocal relationships using cross-sectional data and therefore encourage future longitudinal research employing existing variables with additional technology variables.

## Data availability

### Underlying data

Figshare: Dataset of Questionnaire Result from the respondents of Transformational Entrepreneurship, Readiness to change, Psychological Capital, and Employee Performance. https://doi.org/10.6084/m9.figshare.14267393.v4 ( Gunawan et al., 2021a).

This project contains the following underlying data:

- Questionnaire results from 257 Indonesian bank branch managers.

### Extended data

Figshare: List of questions and descriptions of the questionnaire - Transformational Entrepreneurship. https://doi.org/10.6084/m9.figshare.15091182.v3 ( Gunawan et al., 2021b).

This project contains the following extended data:

- A copy of the questionnaire
- Data coding key

Figshare: The Respondent characteristics of Transformational Entrepreneurship case of a state-owned bank in Indonesia. https://doi.org/10.6084/m9.figshare.14731905.v1 ( Gunawan, et al., 2021c).

This project contains the following extended data:

- Profile of respondents

Data are available under the terms of the Creative Commons Attribution 4.0 International license (CC-BY 4.0).
