# Supplementary material for: Transformational entrepreneurship and its effect on readiness for change, psychological capital, and employee performance: evidence from an Indonesian bank
Source: F1000Res. 2021 Sep 3;10:887. [Version 1] doi: 10.12688/f1000research.52480.1 (PMC8591521; doi:10.12688/f1000research.52480.1)
Supplement: Peer review history

# Peer review: Reviewer response for version 1

**DOI:** 10.5256/f1000research.55763.r93537

**Published:** 2022-01-04

**Authors:** Paul Jones

**Affiliations:** 1 School of Management, Swansea University, Swansea, UK

This study offers an interesting analysis of transformational entrepreneurship in the context of change, psychological capital and employee performance in a developing world banking setting. The literature on transformational literature remains limited especially in a developing world context. So this study represents a welcome addition. This study is generally well executed and draws upon a decent sample of 250 respondents. The study is well written in the appropriate language and draws on a detailed and critical literature review. The study method is well executed using the appropriate method. The study would benefit from a distinct conclusion section which recognises the developing world context and its significance on the results.

Is the work clearly and accurately presented and does it cite the current literature?

Yes

If applicable, is the statistical analysis and its interpretation appropriate?

Yes

Are all the source data underlying the results available to ensure full reproducibility?

No

Is the study design appropriate and is the work technically sound?

Yes

Are the conclusions drawn adequately supported by the results?

Yes

Are sufficient details of methods and analysis provided to allow replication by others?

Yes

Reviewer Expertise:

Transformational Entrepreneurship, Entrepreneurship, small business management

I confirm that I have read this submission and believe that I have an appropriate level of expertise to confirm that it is of an acceptable scientific standard, however I have significant reservations, as outlined above.

# Peer review: Reviewer response for version 1

**DOI:** 10.5256/f1000research.55763.r98858

**Published:** 2021-11-12

**Authors:** Victor Atiase

**Affiliations:** 1 Leicester Castle Business School, De Montfort University, Leicester, UK

Transformational entrepreneurship is still an emerging area and I commend the authors for undertaking this research. However, I believe transformational entrepreneurship is better measured at the country level rather than the firm level. This is because the major goal of TE is scaling up to create mutually beneficial opportunities for everyone in the economy. Issues such as heuristics, systemic and holistic approaches are better identified using country-wide data rather than firm-level data. I, therefore, think the researchers should be measuring transformational leadership which is much more related to the firm rather than TE.

 Measuring TE from the perspective of state-owned banks is just one side of the coin. What are the private sector players, regulators, technological drivers etc?

 However, since this is still an emerging area, I support this publication and I hope the authors will take this further.

Is the work clearly and accurately presented and does it cite the current literature?

Yes

If applicable, is the statistical analysis and its interpretation appropriate?

Yes

Are all the source data underlying the results available to ensure full reproducibility?

Yes

Is the study design appropriate and is the work technically sound?

Yes

Are the conclusions drawn adequately supported by the results?

Yes

Are sufficient details of methods and analysis provided to allow replication by others?

Yes

Reviewer Expertise:

Entrepreneurial Finance

I confirm that I have read this submission and believe that I have an appropriate level of expertise to confirm that it is of an acceptable scientific standard.

# Peer review: Reviewer response for version 1

**DOI:** 10.5256/f1000research.55763.r97563

**Published:** 2021-10-22

**Authors:** Andreas Walmsley

**Affiliations:** 1 Plymouth Marjon University, Plymouth, UK

I like the focus of the paper and believe it should be indexed. It was, on the whole, written clearly and there is some reasonable grounding in the literature. The notion of transformational entrepreneurship is receiving increased interest from scholars but there is still some way to go before we can talk of a substantial body of literature in this area and so on this basis I think the paper does make a valuable contribution to an emerging area of research. This is also however my main area of critique, i.e. specifically the operationalisation of transformational entrepreneurship should be clarified if the paper’s contribution is to be fully realised. If, as the authors argue, TE impacts certain outcome variables the strength of these claims rest on the validity of the TE construct. Unfortunately, it remains unclear how specifically the construct has been operationalised. The authors mention some sources, which are all relevant to defining TE, but as far as I am aware these studies do not operationalise the concept for statistical purposes. Furthermore, because the accompanying dataset was unavailable to me via the links provided I was unable to ascertain the precise nature of the TE as operationalised in the study. Whether I, or other reviewers for that matter, agree with the operationalisation of TE is less important than being able to determine how exactly the construct has been operationalised. Perhaps the authors could ensure this is clarified prior to indexing?

Is the work clearly and accurately presented and does it cite the current literature?

Yes

If applicable, is the statistical analysis and its interpretation appropriate?

Yes

Are all the source data underlying the results available to ensure full reproducibility?

No

Is the study design appropriate and is the work technically sound?

Yes

Are the conclusions drawn adequately supported by the results?

Yes

Are sufficient details of methods and analysis provided to allow replication by others?

Partly

Reviewer Expertise:

Entrepreneurship, entrepreneurship education

I confirm that I have read this submission and believe that I have an appropriate level of expertise to confirm that it is of an acceptable scientific standard, however I have significant reservations, as outlined above.

# Peer review: Reviewer response for version 1

**DOI:** 10.5256/f1000research.55763.r95755

**Published:** 2021-10-15

**Authors:** Gideon Maas

**Affiliations:** 1 International Centre for Transformational Entrepreneurship, Coventry University, Coventry, UK

The focus of this article is on transformational entrepreneurship. There is an active debate on the impact transformational entrepreneurship can play to support sustainable socio-economic development globally. This article contributes to this debate, where there are probably more questions than answers currently, by focusing specifically on the direct and indirect effects of transformational entrepreneurship on readiness for change, psychological capital and employee performance, and how the effects to readiness for change and psychological capital influence employee performance. 

 This article is contributing significantly to the debate on transformational entrepreneurship. I am looking forward to seeing what the impact of this study is on different economic sectors in future.

Is the work clearly and accurately presented and does it cite the current literature?

Yes

If applicable, is the statistical analysis and its interpretation appropriate?

Yes

Are all the source data underlying the results available to ensure full reproducibility?

Yes

Is the study design appropriate and is the work technically sound?

Yes

Are the conclusions drawn adequately supported by the results?

Yes

Are sufficient details of methods and analysis provided to allow replication by others?

Yes

Reviewer Expertise:

Entrepreneurship; family businesses

I confirm that I have read this submission and believe that I have an appropriate level of expertise to confirm that it is of an acceptable scientific standard.
